# Opportunities for selective reporting of harms in randomized clinical trials: Selection criteria for non-systematic adverse events

**DOI:** 10.1186/s13063-019-3581-3

**Published:** 2019-09-05

**Authors:** Evan Mayo-Wilson, Nicole Fusco, Hwanhee Hong, Tianjing Li, Joseph K. Canner, Kay Dickersin

**Affiliations:** 1 Department of Epidemiology and Biostatistics, Indiana University School of Public Health-Bloomington, 1025 E 7th St, #179D, Bloomington, IN 47405 USA; 2 Department of Mental Health, Johns Hopkins Bloomberg School of Public Health, 624 N Broadway, Hampton House, Baltimore, MD 21205 USA; 3 Department of Surgery, Johns Hopkins School of Medicine, 600 North Wolfe Street, Blalock 1202, Baltimore, MD 21287 USA

**Keywords:** Harms, Adverse events, Clinical trials, Reporting bias, Selective outcome reporting, Data sharing, Trial registration

## Abstract

**Background:**

Adverse events (AEs) in clinical trials may be reported in multiple sources. Different methods for reporting adverse events across trials or across sources for a single trial may produce inconsistent information about the adverse events associated with interventions.

**Methods:**

We compared the methods authors use to decide which AEs to include in a particular source (i.e., “selection criteria”), including the number of different types of AEs reported (i.e., rather than the number of events). We compared sources (e.g., journal articles, clinical study reports (CSRs)) of trials for two drug-indications—gabapentin for neuropathic pain and quetiapine for bipolar depression. Electronic searches were completed in 2015. We identified selection criteria and assessed how criteria affected AE reporting.

**Results:**

We identified 21 gabapentin and 7 quetiapine trials. We found 6 gabapentin CSRs and 2 quetiapine CSRs, all written by drug manufacturers. All CSRs reported all AEs without applying selection criteria; by comparison, no other source reported all AEs, and 15/68 (22%) gabapentin sources and 19/48 (40%) quetiapine sources reported using selection criteria. Selection criteria greatly affected the number of AEs reported. For example, 67/316 (21%) AEs in one quetiapine trial met the criterion “occurring in ≥2% of participants in any treatment group,” while only 5/316 (2%) AEs met the criterion “occurring in ≥10% of quetiapine-treated patients and twice as frequent in the quetiapine group as the placebo group.”

**Conclusions:**

Selection criteria for reporting AEs vary across trials and across sources for individual trials. If investigators do not pre-specify selection criteria, they might “cherry-pick” AEs based on results. Even if investigators pre-specify selection criteria, selective reporting will produce biased meta-analyses and clinical practice guidelines. Data about all AEs identified in clinical trials should be publicly available; however, sharing data will not solve all the problems identified in this study.

**Electronic supplementary material:**

The online version of this article (10.1186/s13063-019-3581-3) contains supplementary material, which is available to authorized users.

## Introduction

The U.S. Food and Drug Administration (FDA) and other regulators may approve drugs and biologic agents for marketing when the results of randomized clinical trials indicate that their potential benefits outweigh their potential harms, which are often called “adverse events” (AEs). In clinical trials, AEs can be collected non-systematically when participants report them spontaneously to investigators or in response to open-ended questions such as “have you noticed any symptoms since your last examination?” (Table 1) [1–4]. Specific AEs can also be collected systematically by assessing their presence or absence using the same method for all participants in a trial (e.g., clinical examination, questionnaires, and medical instruments [1, 2]). Previous studies have described non-systematic AEs as “passively collected” and systematic AEs as “proactively collected” [1, 2]. AEs are categorized as “serious” when they lead to or prolong hospitalization, cause death, or disrupt normal life functions [4, 5].

**Table 1:** Terms related to adverse events (AEs)

Term	Definition
Adverse event (AE)	The International Conference on Harmonisation (ICH) defines an “adverse event” as “any untoward medical occurrence in a patient or clinical investigation subject administered a pharmaceutical product and which does not necessarily have to have a causal relationship with this treatment” [33]. The U.S. Food and Drug Administration (FDA) and other regulators use this definition [4, 5].
Non-systematic adverse events	According to The Final Rule [1, 2], “‘non-systematic assessment’ relies on the spontaneous reporting of adverse events, such as unprompted self-reporting by participants”. Non-systematic adverse events may be collected by asking questions such as “Have you noticed any symptoms since your last examination?”.
Result	In the Multiple Data Sources (MUDS) study, a “result” is a numerical contrast between a treatment and comparison arm (e.g., relative risk, mean difference).
Serious adverse events	The ICH defines a “serious adverse event” as that which “results in death, is life-threatening, requires inpatient hospitalisation or prolongation of existing hospitalisation, results in persistent or significant disability/incapacity, or is a congenital anomaly/birth defect” [33]. The FDA and other regulators use this definition [4, 5].
Systematic adverse events	According to The Final Rule [1, 2], “systematic assessment’ involves the use of a specific method of ascertaining the presence of an adverse event (e.g., the use of checklists, questionnaires, specific laboratory tests at regular intervals)”. Like a potential benefit of treatment, a systematic AE can be defined using five elements: (1) domain, (2) specific measurement, (3) specific metric, (4) method of aggregation, and (5) time-point [34]. For example, “proportion of participants with 50% change from baseline to 8 weeks on the Young Mania Rating Scale total score.”
Terms related to sources	
Clinical study report (CSR)	A comprehensive document, often created by a pharmaceutical manufacturer for submission to a regulator, detailing the design, methods, analyses, and results of a study. Appendices sometimes contain tables of individual patient data, also called “patient data listings”, and study protocols [35].
Clinical study report synopsis (CSR-synopsis)	A document that summarizes the information contained in a clinical study report. Clinical study report-synopses are much shorter than clinical study reports; the two clinical study report-synopses we examined were each 13 pages in length.
Individual patient data (IPD)	A table or database in which each record contains data for a single participant [35].
Non-public sources	In the MUDS study, non-public sources include individual patient data, clinical study reports, and clinical study report-synopses.
Public sources	In the MUDS study, public sources include journal articles, conference abstracts, commentaries, posters, trial registrations and associated results, and medical reviews and statistical reviews written by the FDA.

Physicians, patients, and policy makers rely on systematic reviews and clinical practice guidelines to make medical decisions. Syntheses of clinical trial findings should include all available evidence; however, they are often based on AE information reported in public sources, such as journal articles (Table 1) [6]. Many systematic reviews that plan to synthesize AEs ultimately do not address AEs [7]. By contrast, regulators have access to non-public sources of trial information. Although previously non-public sources are becoming available for some trials [8–10], including through data sharing services such as Vivli [11], Yale Open Data Access (YODA) [12, 13], and clinicalstudydatarequest.com, clinical study reports (CSRs) and individual patient datasets (IPD) remain unavailable for many trials. Consequently, the results of many trials are available only in public sources [14, 15], which are often incomplete [15–24]. Reporting bias, which is the selective reporting of research results, occurs when reporting is influenced by the nature of the results (e.g., the direction, magnitude, or statistical significance of the results). Reporting bias may lead to overestimating potential benefits of an intervention [15–20] and underestimating potential AEs [22, 25–32].

There is little evidence about the methods trialists use to select AEs for reporting, and there is little evidence about whether those methods contribute to reporting bias. Previous guidance for reporting adverse events has discouraged vague statements about AEs [36, 37], and the Consolidated Standards of Reporting Trials (CONSORT) Extension for Harms discourages authors from reporting AEs above frequency thresholds (e.g., > 10%) [38]. Nonetheless, there is little evidence about how different trials and different sources for a single trial report AEs. The objectives of this study were to (1) compare selection criteria for reporting non-systematic AEs (i.e., the methods authors use to decide which AEs to include in a particular source as illustrated in Table 2, (2) examine how different selection criteria could affect AE reporting, and (3) assess how different selection criteria could impact meta-analyses of AEs.

**Table 2:** Examples of selection criteria, with each component identified in bold

**Two selection criteria (i.e., numerical threshold and participant group):**	
Adverse events are reported if they occurred in **≥ 5%** of **participants in any intervention group**.	
Adverse events are reported if they occurred in **≥ 2%** of **participants receiving gabapentin**.	
**Three selection criteria:**	
Adverse events are reported if they occurred in **≥ 2%** of **participants receiving gabapentin** and if they occurred **at least twice as frequently** in participants receiving gabapentin, compared with participants receiving placebo.	

## Methods

This analysis is a sub-study of the Multiple Data Sources (MUDS) study. MUDS was a methodologic study, the overall objective of which was to examine multiple data sources about trials and to determine whether the information source could affect the conclusions of systematic reviews and meta-analyses. The published protocol and amendments [24, 39] describe the study methods. Briefly, we searched for public and non-public sources (see definitions in Table 1) and requested additional non-public sources (e.g., CSRs that they had not been disclosed previously) from the companies that manufacture gabapentin and quetiapine as described elsewhere [22, 39, 40]. Electronic searches included the Cochrane Central Register of Controlled Trials (CENTRAL), PubMed, Embase, LILACS, and CINAHL through 2 March 2015 for gabapentin trials and through 26 January 2015 for quetiapine trials. For trial registrations, we searched the International Clinical Trials Registry Platform Search Portal and ClinicalTrials.gov through 10 October 2014.

### Eligible trials and sources

Eligible studies were parallel randomized clinical trials that compared either gabapentin for neuropathic pain or quetiapine for bipolar depression, with placebo in adults. We excluded open-label and crossover trials. We selected these case studies because we had access to both public and non-public sources for some of the eligible trials.

The MUDS study included 21 gabapentin trials (80 sources, including 6 IPD) and 7 quetiapine trials (52 sources, including 2 IPD). We excluded IPD from this sub-study because IPD did describe methods for analyzing data and because the CSRs included aggregate results that were consistent with the IPD. Thus, we included 68 public sources and 6 non-public sources (all CSRs) for gabapentin trials and we included 46 public sources and 4 non-public sources (2 CSRs, 2 CSR-synopses) for quetiapine trials. All studies were reported in one or more public sources, except one gabapentin trial that was not reported in any public source.

### Data extraction

From each source of each eligible trial, two investigators independently extracted data using the open access Systematic Review Data Repository (SRDR; http://srdr.ahrq.gov/) and resolved differences by discussion. We extracted results (i.e., the number or proportion of participants who experienced AEs) that were reported for each trial; we did not extract results of analyses that pooled multiple trials.

We classified each AE as “systematic” if its presence or absence was recorded for every participant and assessed using specific measurement tools (e.g., questionnaires, checklists, laboratory tests, or clinical examinations); we classified all other AEs as “non-systematic” (Table 1). For each non-systematic AE, we extracted the name of the event (e.g., “dizziness”, “headache”) and the numerical results closest to 8 and 18 weeks (e.g., proportion of participants in each group who experienced the AE at 6 weeks). We selected 8 weeks (time window 4–13 weeks) a priori as the minimum clinically important time period, and we selected 18 weeks (time window 14–22) because we expected to find some available follow-up data in this time window. We also looked for data at 27 weeks (time window 23–31 weeks) and for longer times, but we did not find trials that reported AEs after the 8 and 18 week time windows.

We extracted the names of non-systematic AEs even when numerical results were not reported; for example, if a source reported that “the most common AEs were dizziness and headache,” we extracted that dizziness and headache were reported and we entered the numerical results as missing data [22].

We extracted the methods from each source that authors said they used to select AEs for inclusion in the source, which we refer to as “selection criteria” or we recorded that the selection criteria were not reported (Table 2). Although we found evidence that potential benefits and systematic AEs are reported selectively [23], we did not find that systematic AEs were reported based on selection criteria as described in this sub-study. Thus, we describe selection criteria for non-systematic AEs only.

### Comparing non-systematic AE selection criteria sources and trials

We compared the selection criteria for non-systematic AEs reported across sources for each trial (see examples in Table 2). We recorded three pre-specified components of non-systematic AE selection criteria:

- Numerical threshold: a cutoff for reporting the number or proportion of participants who experienced a non-systematic AE (e.g., ≥ 5% of participants).
- Participant group: the group(s) in a trial that must have experienced an AE (e.g., participants in the test intervention group, all participants in the trial).
- Difference-between-groups: a cutoff for reporting a difference in the number or proportion of participants who experienced an AE, comparing one participant group with another (e.g., more frequent in the test intervention group compared with the placebo group).

### Applying AE selection criteria to data in individual trials

To assess how different selection criteria might affect which and how many non-systematic AEs would be reported, we combined each of the numerical thresholds we observed (*N* = 5) with each of the participant-group criteria (*N* = 3) and difference-between-groups criteria we observed (*N* = 3) to create 45 combined selection criteria.

We observed some selection criteria in both gabapentin and quetiapine trials, and we observed other selection criteria in gabapentin trials only or in quetiapine trials only. Although we analyzed only numerical thresholds, participant groups, and differences between groups that we observed in eligible trials, we never observed some of these combined selection criteria in any trial.

Using all non-systematic AEs reported in each CSR, we then applied each of 45 combined selection criteria to the data found in CSRs for six gabapentin trials and two quetiapine trials. We estimated the number of AEs (i.e., the different types of AEs rather than the number of events) that would have been reported according to each combined criterion.

### Statistical methods

We calculated descriptive statistics (i.e., counts) using Stata 14 [41].

## Results

### AE selection criteria in public and non-public sources

We identified selection criteria only in public sources and in CSR-synopses. CSRs, which included trial protocols, did not report using selection criteria. Based on the information in CSRs, including trial protocols, CSRs appeared to include information about all observed AEs. CSRs did not describe which selection criteria would be used to report AEs in other sources, such as journal articles; thus, we found no scientific rationale or evidence that selection criteria used in other sources were pre-specified [35].

In public sources and CSR-synopses we identified:

- Five different numerical thresholds, including four in gabapentin sources (1%, 2%, 3%, and 5%) and two in quetiapine sources (5% and 10%);
- Three different participant groups; all three participant groups were included in both gabapentin and quetiapine sources. These were (1) participants in any single intervention group, (2) participants receiving the test intervention (i.e., gabapentin or quetiapine), and (3) all trial participants (i.e., frequency in the combined intervention groups); and
- Three different differences between groups, including two in gabapentin sources (no difference in frequency, frequency “higher” in the gabapentin group compared with the placebo group) and two in quetiapine sources (no difference in frequency, frequency “at least twice as high” in the quetiapine group).

**Fig. 1:**
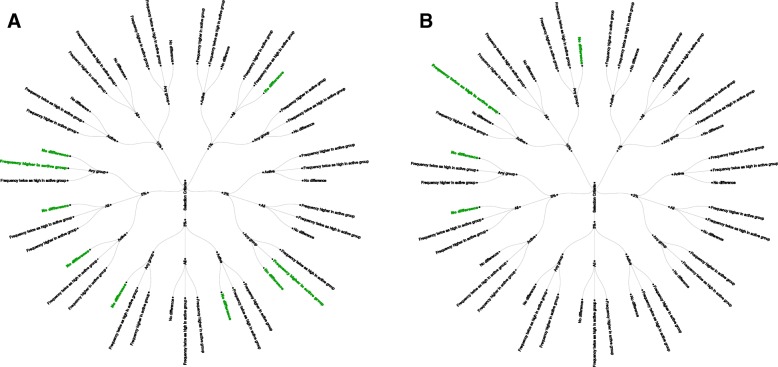
Observed components of selection criteria. Selection criteria that we applied in sources about gabapentin (**a**) and quetiapine (**b**). Shown are all possible combinations of selection criteria. Each of the three rings of the circle represents a different component of the selection criteria: numerical threshold, participant group, and difference in frequency threshold. Green text indicates selection criteria reported in public sources. All = all participants combined across groups; Any group = participants in a particular intervention group; Active = participants in the active intervention group (i.e., gabapentin or quetiapine).

We observed 9 combined selection criteria in gabapentin trials and 4 combined selection criteria in quetiapine trials, all in public sources and CSR-synopses (Fig. 1). Each of the selection criteria that we observed included a numerical threshold (albeit different numerical thresholds). Selection criteria did not always include information about participant group or differences between groups; when they did include this information, selection criteria often differed.

When multiple sources described the same trial(s), selection criteria sometimes varied across sources (Additional file 1). Sources about the same trial were sometimes discrepant; in 2/7 (29%) trials with multiple sources that reported selection criteria, we found different sources reported different AEs (Additional file 1). In one additional trial, we found a public source that did not report AEs found in the CSR that should have been reported in the public source according to the selection criteria described in the public source (Additional file 1).

### Number of trials and sources providing AE selection criteria

**Fig. 2:**
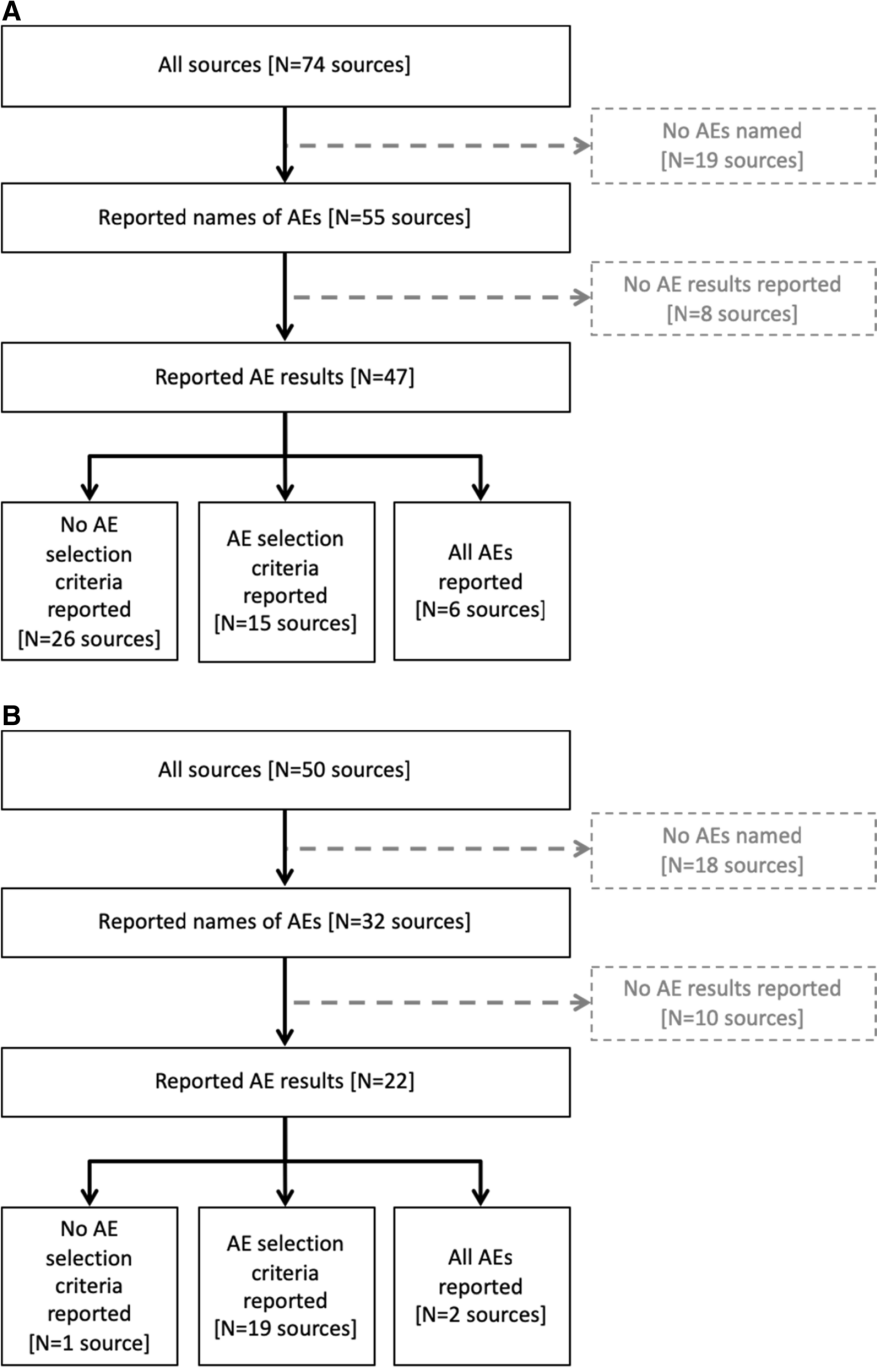
Reported adverse events (AEs) and adverse event selection criteria. **a** Gabapentin. Six clinical study reports (CSRs) we identified appeared to report all AEs. All other sources in this figure are public sources. **b** Quetiapine. Two CSRs we identified appeared to report all AEs. Two CSR-synopses reported AE results and AE selection criteria. All other sources in this figure are public sources

Although we found no trial with a public source that included all non-systematic AEs (Fig. 2), 47/74 (64%) gabapentin sources and 22/50 (44%) quetiapine sources reported some AE results. Of the sources that did report AE results, 15/47 (32%) gabapentin sources and 19/22 (86%) quetiapine sources reported the selection criteria used.

### Selection criteria affect AE reporting

**Fig. 3:**
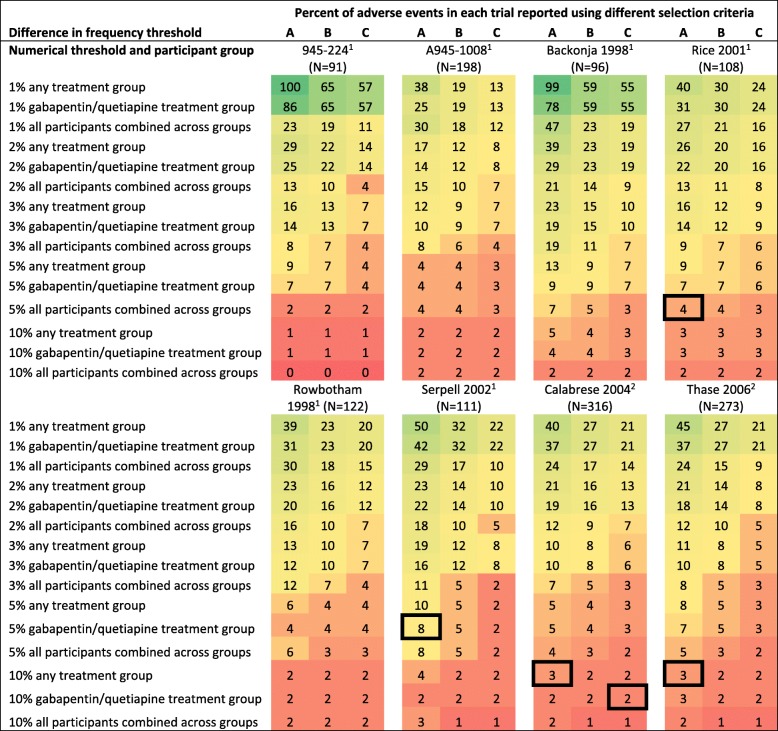
Percentage of adverse events (AEs) that would be reported using different selection criteria. We applied 45 different selection criteria to AEs in each of the eight trials for which we identified a clinical study report (CSR). To illustrate the potential variation in reported non-systematic AEs for each trial, we calculated the percentage of AEs that would be reported using each selection criterion, and we colored the figure using a heat map, with green representing the most AEs and red representing the fewest AEs. Squares outlined in black represent the selection criteria used in at least one source about that trial (e.g., a source describing Serpell 2002 reported AEs that occurred in ≥ 5% of participants in the gabapentin trial). Some trials did not have any sources that described the selection criteria (e.g., 945–224). **A** = no difference in frequency threshold; **B** = higher frequency in gabapentin/quetiapine than in placebo; **C** = frequency in gabapentin/quetiapine at least twice as high as placebo. ^**1**^Gabapentin trial; ^**2**^Quetiapine trial

We examined the impact of selection criteria on reporting AEs using all trials for which we had a CSR (Fig. 3). By applying the 45 selection criteria to the AEs reported in eight CSRs, we determined that selection criteria could have a meaningful impact on the number of different AEs that would be reported in other sources (Fig. 3). For example, there were 316 different AEs described in the CSR for Calabrese 2004 [42]. While 126/316 (40%) AEs met the selection criterion “occurring in ≥1% of participants in any treatment group,” only 5/316 (2%) AEs met the selection criterion “occurring in ≥10% of quetiapine-treated patients and twice as frequent in the quetiapine group as the placebo group.” All public sources about Calabrese 2004 that reported selection criteria indicated that they described all AEs occurring in ≥ 10% of quetiapine-treated patients and twice as frequently in the quetiapine group compared with the placebo group.

## Discussion

In this study, we examined how reports of clinical trials use selection criteria for reporting non-systematic AEs. We found that public sources and CSR-synopses applied selection criteria while CSRs reported all AEs; consequently, most AEs and serious AEs were not reported in public sources. In public sources, every combined selection criterion we found included a numerical threshold; however, numerical thresholds were not consistent across trials or across sources for individual trials. Some selection criteria also included requirements related to the participant group and the difference between groups.

We found no evidence in public sources (e.g., journal articles) or non-public sources (e.g., CSRs) that any trial used pre-specified selection criteria. We assume that public sources reporting a small number of AEs used selection criteria; however, many public sources did not describe the selection criteria they used. Among public sources that did *not* describe selection criteria, we could not determine why the authors reported some non-systematic AEs but did not report other non-systematic AEs. Even when selection criteria were described, public sources did not explain why the authors applied those selection criteria (e.g., following a pre-specified statistical analysis plan). Even if investigators could identify which AEs or groups of AEs were most important in a given trial, different selection criteria across trials would make it impossible to ensure unbiased reporting and to conduct unbiased syntheses.

When we applied various selection criteria to eligible trials, we observed meaningful differences in the number of AEs that would be reported. Trialists might have used selection criteria to identify what they consider the most important AEs; however, we are unaware of any consensus about which AEs are most important for these drugs and conditions. We found no evidence that patients or clinicians were involved in deciding which AEs would be reported in public sources. Instead, it appears that public sources included the most common AEs. Trialists could have performed analyses similar to ours in which they applied different selection criteria and then decided *post hoc* which selection criteria would allow them to report, or not report, particular AEs. In a previous study, we found that trialists did not “group” AEs for reporting [22], which would be another method to consolidate AEs for reporting that has been advocated elsewhere [37]; however, grouping AEs can also disguise important AEs by combining them with less important AEs [43].

Evidence syntheses (e.g., systematic reviews, clinical practice guidelines) could help identify rare AEs if all observed AEs were available for all trials [44]; however, rare AEs cannot be identified when clinical trials report only those AEs occurring above numerical thresholds. Just as selectively reporting potential benefits based on quantitative results leads to biased meta-analyses [45–48], reporting AEs based on trial results will necessarily lead to biased overall estimates because only “positive” signals will be available. Selection criteria make it impossible for systematic reviewers and meta-analysts to accurately assess the AEs caused by medical interventions.

We found that no public source reported all AEs for any trial. Only serious AEs and AEs occurring in more than 5% of participants are required to be reported in www.ClinicalTrials.gov according to the Food and Drug Administration Amendments Act of 2007 (FDAAA) [1, 49]. Doctors and patients often rely on post-marketing surveillance studies to identify rare AEs, including serious AEs, yet these studies lack comparison groups that are present in clinical trials. It might be possible to detect rare AEs in clinical trials and to calculate between-group differences, if investigators would not use numerical thresholds to determine which AEs to report. Investigators must ensure that for common AEs, such as headache, data are collected consistently across intervention groups so that proportions in each group can be compared. This is difficult when AEs are collected non-systematically. Although systematically collecting AEs would result in more trustworthy and usable information about effects between groups [22, 23], it is not always possible, or even desirable, to anticipate which AEs patients will experience.

Authors have previously discouraged the use of selection criteria [36–38]; however, trials would have to report hundreds or thousands of AEs if selection criteria were not used. Making CSRs and equivalent reports for non-industry trials public, and grouping AEs for analysis and reporting, would partially address the problem of reporting many AEs in journal articles and other public sources. A complete solution to the problems we have identified is not obvious.

Multiple sources of public AE information from trials (e.g., journal articles, FDA reviews), which may be written by different authors for different purposes, lead to inconsistent information for patients and physicians [50]. For example, FDA reviewers have access to CSRs that report all AEs that occurred in each trial. FDA reviewers consider pre-clinical data (e.g., pharmacokinetics, animal trials) and apply clinical and statistical judgment when deciding what to report in medical and statistical reviews about new drugs and biologic agents. The FDA and manufacturers also decide what to include in prescribing information (drug “labels”) written for patients and doctors. By comparison, other stakeholders obtain their information about interventions from a variety of sources, and those sources may use different selection criteria for reporting. For example, individual authors and journal editors can decide what to report in journal articles, which vary tremendously. Different selection criteria across reports of clinical trials, including multiple reports of the same trial, lead to inconsistent and confusing information for stakeholders; consistent standards for reporting AEs, and open access to trial information (e.g., CSRs) could help.

Because current methods for selecting AEs lead to biased reporting that will necessarily produce incorrect effect estimates in systematic reviews and clinical practice guidelines, regulators, lawmakers (e.g., the U.S. Congress) and journals could require that trialists report all AEs on www.ClinicalTrials.gov or other registries. When it is not feasible to report all AEs in a given source (e.g., a conference abstract), the source could direct readers to additional information in a registry such as www.ClinicalTrials.gov. FDA and other regulators could make all AEs publicly available. If all AEs are not made available by regulators or by trialists, systematic reviewers and meta-analysts should interpret results with extreme caution and explain the limitations of using only publicly available data.

There is a pressing need to make clinical trial data available to the public, especially CSRs and IPD; however, sharing CSRs and IPD will not solve all problems identified in our research. First, reports describing hundreds of AEs might overwhelm physicians and patients by including “too much” information [51]. Many AEs reported in CSRs and IPD are not intervention-related; selection criteria might have been used to help decision-makers identify AEs that are caused by medical products. Sharing lengthy and confusing reports and datasets could increase the appearance of transparency while actually disguising important information. Second, reanalyzing clinical trial data is time consuming and therefore expensive, and reanalysis should not be necessary to identify AEs. Most decision-makers want trustworthy *summaries* of clinical trials.

To avoid reporting bias, and to avoid overwhelming patients and physicians with information, trials could pre-specify which AEs will be collected and reported in summaries such as journal articles [36]. Core outcome sets are the minimum sets of outcomes to include in trials [52, 53], and they normally focus on the assessment of potential benefits for people seeing treatment for a particular health problem. Because different types of interventions for the same health problem might be associated with different AEs, and because a single intervention might be used to treat several health problems (e.g., quetiapine is used to treat bipolar depression and schizophrenia), core outcome sets focused on the AEs associated with a particular intervention or group of interventions could also improve the comparability of clinical trials. Notably, core outcome sets for AEs would help trialists identify and report those AEs that are most important to patients, not just the AEs that occurred most often [22, 23, 51]. As described elsewhere, systematic assessment of important AEs would produce much more useful information compared with non-systematic assessment [22, 23].

It is a limitation that we included only two drug-indications, and that we had non-public information for a minority of trials in each case study. The use of selection criteria and the extent to which they are pre-specified and consistent might differ across companies and investigators. Nonetheless, the results of this methodological investigation highlight fundamental problems with methods currently used to report AEs that occur in clinical trials.

## Conclusions

Stakeholders require complete AE information to make informed healthcare decisions. Our findings show that systematic reviews and clinical practice guidelines are unlikely to provide accurate effect estimates for AEs because the use of selection criteria results in limited and necessarily biased information about AEs. It may be both difficult and undesirable to include all AEs in journal articles and other public reports, so standards are needed to determine which AEs to include in reports of clinical trials and how to report AEs completely in other public sources such as trial registers [54].

## Additional file

Additional file 1: Comparison of adverse events reported in sources about the same trial. (DOCX 266 kb)

## Data Availability

The datasets generated and analyzed during the current study and the statistical code are available from the Dryad repository at https://datadryad.org/resource/doi:10.5061/dryad.mp26fb1.

## Abbreviations

AE: Adverse event
CSR: Clinical study report
FDA: U.S. Food and Drug Administration
FDAAA: Food and Drug Administration Amendments Act of 2007
IPD: Individual patient dataset
MUDS: Multiple Data Sources Study
SRDR: Systematic Review Data Repository
