# Supplementary material for: Opportunities for selective reporting of harms in randomized clinical trials: Selection criteria for non-systematic adverse events
Source: Trials. 2019 Sep 5;20:553. doi: 10.1186/s13063-019-3581-3 (PMC6728982; doi:10.1186/s13063-019-3581-3)
Supplement: Supplementary file 1 — Comparison of adverse events reported in sources about the same trial. (DOCX 266 kb) [file 13063_2019_3581_MOESM1_ESM.docx]

## Additional file 1: Comparison of adverse events reported in sources about the same trial

Legend:

*This column is “Not applicable” when either (a) the sources did not report any selection criteria or (b) exactly one source reported a criterion, and therefore there are no other sources for comparison.

| **Trial identifier** | **Reported selection criteria** | **No. sources** | **Did sources using the same selection criteria also report the same adverse events?*** | **Differences among sources** |
| --- | --- | --- | --- | --- |
| **Gabapentin for neuropathic pain** | | | | |
| 945-224 | Selection criteria not reported | 5^(1-5)^ | Not applicable^a^ | Not applicable |
|  | All AEs reported | 1^(6)^ | Not applicable^b^ |  |
| A945-1008 | All AEs reported | 1^(7)^ | Not applicable^b^ | Not applicable |
| Arai 2010 | Selection criteria not reported | 2^(8, 9)^ | Not applicable^a^ | Not applicable |
| Backonja 1998 | Selection criteria not reported | 13^(1-5, 10-17)^ | Not applicable^a^ | Not applicable |
|  | All AEs reported | 1^(18)^ | Not applicable^b^ |  |
| Caraceni 2004 | Selection criteria not reported | 3^(19-21)^ | Not applicable^a^ | Not applicable |
| Hahn 2004 | Selection criteria not reported | 1^(22)^ | Not applicable^a^ | Not applicable |
| Hui 2010 | Selection criteria not reported | 2^(23, 24)^ | Not applicable^a^ | Not applicable |
| Irving 2009 | Selection criteria not reported | 1^(25)^ | Not applicable^a^ | Not applicable |
|  | 5% any treatment group | 1^(26)^ | Not applicable^b^ |  |
| Milenkovic 2009 | Selection criteria not reported | 1^(27)^ | Not applicable^a^ | Not applicable |
| Mishra 2012 | Selection criteria not reported | 1^(28)^ | Not applicable^a^ | Not applicable |
| NCT00475904 | 5% any treatment group | 1^(29)^ | Not applicable^b^ | Not applicable |
| Rice 2001 | Selection criteria not reported | 10^(1-5, 30-34)^ | Not applicable^a^ | The CSR^(35)^ included all AEs. Two sources^(36, 37)^ reported data for two AEs that did not occur in ≥5% patients according to the CSR; based on information in the CSR, we believe the public sources reported AEs occurring in 5% of gabapentin-treated patients. |
|  | 5% all patients combined across treatment groups | 2^(36, 37)^ | Yes |  |
|  | All AEs reported | 1^(35)^ | Not applicable^b^ |  |
| Rowbotham 1998 | Selection criteria not reported | 12^(1-5, 30-33, 38-40)^ | Not applicable^a^ | Not applicable |
|  | All AEs reported | 1^(41)^ | Not applicable^b^ |  |
| Sandercock 2012 | Selection criteria not reported | 2^(42, 43)^ | Not applicable^a^ | Not applicable |
|  | 5% any treatment group | 1^(44)^ | Not applicable^b^ |  |
| Sang 2013 | Selection criteria not reported | 10^(45-54)^ | Not applicable^a^ | One source with data for two trials (Sang 2013 and Wallace 2010)^(55)^ reported one AE that was not reported in two sources^(56, 57)^; although the selection criteria differed, the AE reported in the first source^(55)^ met the selection criteria used in the other sources^(56, 57)^. |
|  | 1% all patients combined across treatment groups | 1^(58)^ | Not applicable^b^ |  |
|  | 2% any treatment group | 1^(59)^ | Not applicable^b^ |  |
|  | 2% any treatment group & higher frequency in gabapentin treatment group | 1^(60)^ | Not applicable^b^ |  |
|  | 3% any treatment group | 2^(56, 57)^ | Yes |  |
|  | 3% gabapentin treatment group | 1^(55)^ | Not applicable^b^ |  |
|  | 5% any treatment group & higher frequency in gabapentin treatment group | 1^(61)^ | Not applicable^b^ |  |
| Serpell 2002 | Selection criteria not reported | 8^(1-5, 30, 62, 63)^ | Not applicable^a^ | Not applicable |
|  | 5% gabapentin treatment group | 1^(64)^ | Not applicable^b^ |  |
|  | All AEs reported | 1^(65)^ | Not applicable^b^ |  |
| Simpson 2001 | Selection criteria not reported | 2^(66, 67)^ | Not applicable^a^ | Not applicable |
| Tamez Perez 2000 | Selection criteria not reported | 2^(68, 69)^ | Not applicable^a^ | Not applicable |
| Wallace 2010 | Selection criteria not reported | 10^(45-52, 70, 71)^ | Not applicable^a^ | One source with data for two trials (Sang 2013 and Wallace 2010)^(55)^ reported one AE that was not reported in two sources^(56, 57)^; although the selection criteria differed, the AE reported in the first source^(55)^ met the selection criteria used in the other sources^(56, 57)^. |
|  | 2% any treatment group | 1^(59)^ | Not applicable^b^ |  |
|  | 2% any treatment group & higher frequency in gabapentin treatment group | 1^(60)^ | Not applicable^b^ |  |
|  | 3% any treatment group | 2^(56, 57)^ | Yes |  |
|  | 3% gabapentin treatment group | 1^(55)^ | Not applicable^b^ |  |
|  | 5% any treatment group | 1^(72)^ | Not applicable^b^ |  |
| Yildirim 2003 | Selection criteria not reported | 1^(73)^ | Not applicable^a^ | Not applicable |
| Zepeda Vazquez 2001 | Selection criteria not reported | 1^(74)^ | Not applicable^a^ | Not applicable |
| **Quetiapine for bipolar depression** | | | | |
| Calabrese 2004 | Selection criteria not reported | 15^(75-89)^ | Not applicable^a^ | The CSR^(90)^ included all AEs. Using the selection criteria reported in other sources, the AEs reported in other sources^(91-100)^ were consistent with the data in the CSR. |
|  | 5% any treatment group | 2^(91, 92)^ | Yes |  |
|  | 5% all patients combined across treatment groups | 1^(93)^ | Not applicable^b^ |  |
|  | 10% any treatment group | 2^(94, 99)^ | Yes |  |
|  | 10% quetiapine treatment group & frequency in quetiapine treatment group twice as high as placebo | 5^(95-98, 100)^ | Yes |  |
|  | All AEs reported | 1^(90)^ | Not applicable^b^ |  |
| Gao 2014 | 5% all patients combined across treatment groups | 1^(101)^ | Not applicable^b^ | Not applicable |
|  | 5% any treatment group | 1^(102)^ | Not applicable^b^ |  |
| Li 2014 | Selection criteria not reported | 1^(103)^ | Not applicable^a^ | Not applicable |
|  | 5 most common AEs | 1^(104)^ | Not applicable^b^ |  |
| McElroy 2010 | Selection criteria not reported | 9^(76-81, 91, 105, 106)^ | Not applicable^a^ | Not applicable |
|  | 5% any treatment group | 3^(107-109)^ | Yes |  |
| Suppes 2010 | Selection criteria not reported | 2^(110, 111)^ | Not applicable^a^ | Not applicable |
|  | 5% any treatment group | 1^(112)^ | Not applicable^b^ |  |
| Thase 2006 | Selection criteria not reported | 10^(76-81, 113-116)^ | Not applicable^a^ | The CSR^(117)^ included all AEs. Using the selection criteria reported in other sources, the AEs reported in other sources^(91-95, 118)^ were consistent with the data in the CSR. |
|  | 5% any treatment group | 2^(91, 92)^ | Yes |  |
|  | 5% all patients combined across treatment groups | 1^(93)^ | Not applicable^b^ |  |
|  | 10% any treatment group | 2^(94, 118)^ | Yes |  |
|  | 10% quetiapine treatment group & frequency in quetiapine treatment group twice as high as placebo | 1^(95)^ | Not applicable^b^ |  |
|  | All AEs reported | 1^(117)^ | Not applicable^b^ |  |
| Young 2008 | Selection criteria not reported | 11^(76-81, 107, 119-122)^ | Not applicable^a^ | Not applicable |
|  | 5% any treatment group | 3^(91, 123, 124)^ | Yes |  |

**References**

1. Backonja M, Glanzman RI. Gabapentin dosing for neuropathic pain: evidence from randomized,placebo-controlled clinical trials. Clinical Therapeutics. 2003;25:81-104.

2. Backonja MM, Mutisya E. Dose response to gabapentin across five multicenter trials for neuropathic pain. Annals of Neurology. 2002(Suppl 1):S81.

3. Backonja M-M, Mutisva EM, editors. Gabapentin demonstrates a nonlinear dose-reponse across five multicenter trials for neuropathic pain (Poster). 2002 EFNS Annual Congress; 2002 26-29 October, 2002; Vienna, Austria.

4. Rowbotham MC, Backonja MM, Knapp LE, Yan C, Purcell TJ, Koto E, editors. Gabapentin in the management of neuropathic pain in patients with diabetic peripheral neuropathy, postherpetic neuralgia, and neuropathic pain of varying etiology: Overview of results from 5 placebo-controlled trials (poster). ICMTNP Annual Meeting; 2002.

5. Backonja M, Mutisya EM. Backonja M, Mutisya EM. Review of gabapentin dosing in five placebo‐controlled clinical trials for neuropathic pain. Eur J Neurol. 2002;Suppl 2:191.

6. Parke-Davis. Research Report: 720-04130. A Double-Blind Placebo-Controlled Trial With 3 Doses of Gabapentin for Treatment of Painful Diabetic Neuropathy. Protocol Number: 945-224. 2000 7 February 2000.

7. Pfizer. Final study report: A 15 Week randomized, double-blind, placebo controlled, parallel-group, multicenter study of Neurontin (gabapentin) for eficacy and quality of life in patients with painful diabetic peripheral neuropahty. Protocol A945-1008.; 2005 24 March 2005.

8. Arai YP, Matsubara T, Shimo K, Ushida T, Osuga T, Nishihara M. Low dose gabapenttin as useful adjuvant to opioids for neuropathic cancer pain when combined with low dose imipramine. Anesthesia and Analgesia. 2010;110(3):S365.

9. Arai YC, Matsubara T, Shimo K, Suetomi K, Nishihara M, Ushida T, et al. Low-dose gabapentin as useful adjuvant to opioids for neuropathic cancer pain when combined with low-dose imipramine. Journal of Anesthesia. 2010;24(3):407-10.

10. Slawson D. How effective is gabapentin (Neurontin) in reducing the pain associated with diabetic peripheral neuropathy? Evidence-Based Practice. 1999;2(3):7, 2p.

11. Schiebel NEE, Ebbert J, Margolis K, Backonja M. Gabapentin for painful diabetic neuropathy [3] (multiple letters). JAMA. 1999;282(2):133-4.

12. Backonja MM. Gabapentin monotherapy for the symptomatic treatment of painful neuropathy: a multicenter, double-blind, placebo-controlled trial in patients with diabetes mellitus. Epilepsia. 1999;40 Suppl 6:S57-9; discussion S73-4.

13. Backonja M, Beydoun A, Edwards KR, Schwartz SL, Fonseca V, Hes M, et al. Gabapentin for the symptomatic treatment of painful neuropathy in patients with diabetes mellitus: a randomized controlled trial. JAMA. 1998;280(21):1831-6.

14. Anonymous. Gabapentin for diabetic neuropathy. Nurses' Drug Alert. 1999;23(2):11.

15. Edwards KR, Bannington VT, Hes MS, LaMoreaux LK, Garofalo EA, Koto EM. Gabapentin (Neurontin) for pain associated with diabetic peripheral neuropathy: a double-blind, placebo-controlled study (945-210) [abstract] Neurology. 1998;50:A378-A9.

16. Backonja M, Hes MS, LaMoreaux LK, Garofalo EA, Koto EM, The US Gabapentin Study Group 210, editors. Gabapentin reduces pain in diabetics with painful peripheral neuropathy: results of a double‐blind, placebo controlled trial (945‐210). The 16th Annual Scientific Meeting of the American Pain Society; 1997.

17. Seidl JJ, Slawson JG. Gabapentin for painful diabetic neuropathy. Journal of Family Practice. 1999;48(3):173-4.

18. Parke-Davis. Research Report: 720-03908. A Double-Blind Placebo-Controlled Trial of Gabapentin for Treatment of Painful Diabetic Peripheral Neuropathy. Protocol Number: 945-210. 1998 30 December 1998.

19. Caraceni A, Zecca E, Bonezzi C, Bennett MI. Gabapentin significantly improves analgesia in people receiving opioids for neuropathic cancer pain. Cancer Treatment Reviews. 2005;31(1):58-62.

20. Caraceni A, Zecca E, Bonezzi C, Spigel DR. Improving neuropathic pain due to cancer using gabapentin. Journal of Clinical Outcomes Management. 2004;11(10):620-1.

21. Caraceni A, Zecca E, Bonezzi C, Arcuri E, Yaya Tur R, Maltoni M, et al. Gabapentin for neuropathic cancer pain: a randomized controlled trial from the Gabapentin Cancer Pain Study Group. Journal of Clinical Oncology. 2004;22(14):2909-17.

22. Hahn K, Arendt G, Braun JS, Giesen HJ, Husstedt IW, Maschke M, et al. A placebo-controlled trial of gabapentin for painful HIV-associated sensory neuropathies. Journal of Neurology. 2004;251(10):1260-6.

23. Hui AC, Wong SM, Leung HW, Man BL, Yu E, Wong LK. Gabapentin for the treatment of carpal tunnel syndrome: a randomized controlled trial. Eur J Neurol. 2011;18(5):726-30.

24. Gabapentin for carpal tunnel syndrome: A randomised controlled trial. In: ClinicalTrials.gov [Internet]. Bethesda (MD): National Library of Medicine (US). 2000 [cited 26 May 2015]. Available from: <https://clinicaltrials.gov/ct2/show/NCT00137735>. NLM Identifier: NCT00137735 [Internet].

25. Jensen MP, Chiang YK, Wu J. Assessment of pain quality in a clinical trial of gabapentin extended release for postherpetic neuralgia. Clinical J Pain. 2009;25(4):286-92.

26. Irving G, Jensen M, Cramer M, Wu J, Chiang YK, Tark M, et al. Efficacy and tolerability of gastric-retentive gabapentin for the treatment of postherpetic neuralgia: results of a double-blind, randomized, placebo-controlled clinical trial. Clinical J Pain. 2009;25(3):185-92.

27. Milenkovic T, Percan V, Petrovski G, Ahmeti I, Mishevska Jovanovska S. Management of painful diabetic neuropathy with gabapenthin. J Diabetes Complicat. 2009;1:A202-A3.

28. Mishra S, Bhatnagar S, Goyal GN, Rana SP, Upadhya SP. A comparative efficacy of amitriptyline, gabapentin, and pregabalin in neuropathic cancer pain: a prospective randomized double-blind placebo-controlled study. American Journal of Hospice & Palliative Care. 2012;29(3):177-82.

29. Corporation E. A Phase 3 Multicenter, Randomized, Double-Blind, Placebo-Controlled Study of the Safety and Efficacy of Gabapentin Extended Release (G-ER) Tablets in the Treatment of Patients With Postherpetic Neuralgia. In: ClinicalTrials.gov [Internet]. Bethesda (MD): National Library of Medicine (US) 2000 [cited 26 May, 2015]. Available from: <https://clinicaltrials.gov/ct2/show/NCT00475904>. NLM Identifier: NCT00475904.

30. Parsons B, Tive L, Huang S. Gabapentin: a pooled analysis of adverse events from three clinical trials in patients with postherpetic neuralgia. The American journal of geriatric pharmacotherapy. 2004;2(3):157-62.

31. Food and Drug Administration (Center for Drug Evaluation and Research). Statistical Review NDA 21-397. 2002.

32. Food and Drug Administration (Center for Drug Evaluation and Research). Medical Review NDA 21-397. 2002.

33. Anonymous. First approval of oral treatment for postherpetic neuralgia. Formulary. 2002;37(7):335.

34. Rice AS, Maton S. Reply to Comment on "Rice ASC, Maton S, the Postherpetic Neuralgia Study Group (UK), gabapentin in postherpetic neuralgia: a randomized, double blind, placebo-controlled study". Pain.96(3):411-2.

35. Parke-Davis. Research Report: 430-00124. A double-blind placebo controlled trial of gabapentin for the treatment of post herpetic neuralgia. Protocol Number: 945-430-295 (NN 025). 2000 3 April 2000.

36. Rice ASC, Maton S. Gabapentin in postherpetic neuralgia: A randomised, double blind and placebo controlled study. [Spanish]. Revista de la Sociedad Espanola del Dolor. 2002;9(2):61-73.

37. Rice AS, Maton S. Gabapentin in postherpetic neuralgia: a randomised, double blind, placebo controlled study. Pain. 2001;94(2):215-24.

38. Gabapentin for postherpetic neuralgia. Nurses' Drug Alert. 1999;23(2):11-2.

39. Slawson D. How effective and safe is gabapentin (Neurontin) for reducing the pain of postherpetic neuralgia? Evidence-Based Practice. 1999;2(3):7-8, 2p.

40. Rowbotham M, Harden N, Stacey B, Bernstein P, Magnus-Miller L. Gabapentin for the treatment of postherpetic neuralgia: a randomized controlled trial. JAMA. 1998;280(21):1837-42.

41. Parke-Davis. Research Report: 995-00070. Double-blind, randomized, placebo-controlled, parallel groups, multi-center trial to determine the efficacy and safety of Neurontin (gabapentin) in subjects with peripheral neuropathy (post-herpetic neuralgia). Protocol Number: 945-211. 1998 29 December 1998.

42. Depomed. A multicenter, randomized, double-blind, placebo-controlled study of the safety and efficacy of gabapentin extended release (G-ER) tablets in the treatment of patients with painful diabetic peripheral neuropathy. In: ClinicalTrials.gov [Internet]. Bethesda (MD): National Library of Medicine (US) 2000 [cited 26 May, 2015]. Available from: <https://clinicaltrials.gov/ct2/show/NCT00712439>. NLM Identifier: NCT00712439.

43. Sandercock D, Cramer M, Wu J, Chiang YK, Biton V, Heritier M. Gabapentin extended release for the treatment of painful diabetic peripheral neuropathy: efficacy and tolerability in a double-blind, randomized, controlled clinical trial. Diabetes Care. 2009;32(2):e20.

44. Sandercock D, Cramer M, Biton V, Cowles VE. A gastroretentive gabapentin formulation for the treatment of painful diabetic peripheral neuropathy: efficacy and tolerability in a double-blind, randomized, controlled clinical trial. Diabetes Research and Clinical Practice. 2012;97(3):438-45.

45. Kantor D, Mathis JT, Rauck RL, Irving G, Sweeney M, Vanhove GF. Integrated analysis of efficacy of a once-daily gastroretentive formulation of gabapentin in patients with postherpetic neuralgia who are at least 75 years old. J Gen Intern Med. 2012;27:S231.

46. Smith W, Rauck R, Irving G, Wallace M, Vanhove G, Sweeney M. Integrated analysis of efficacy and safety of a once-daily gastroretentive formulation of gabapentin in patients with postherpetic neuralgia. J Am Pharm Assoc. 2012;52(2):263.

47. Rauck R, Irving G, Wallace M, Vanhove G, Sweeney M. Integrated analysis of efficacy and safety of a once-daily gastroretentive formulation of gabapentin in patients with postherpetic neuralgia. Neurology. 2012;78(1).

48. Rauck R, Irving G, Wallace MS, Vanhove GF, Sweeney M. Integrated analysis of efficacy and safety of a once-daily gastroretentive formulation of gabapentin in patients with postherpetic neuralgia Pain Pract. 2012;12:119.

49. Jensen M, Rauck R, Irving G, Sweeney M, Vanhove G. Onset of treatment response to a once-daily gastroretentive formulation of gabapentin in patients with postherpetic neuralgia. J Pain. 2012;13(4 suppl. 1):S70.

50. Sweeney M, Misha B, Wallace M, Freeman R. Effect of a once-daily gastroretentive formulation of gabapentin on the Brief Pain Inventory Scale scores in patients with postherpetic neuralgia. J Pain. 2012;13(4 suppl. 1):S71.

51. Backonja M, Wallace MS, Freeman R, Sweeney M. Effect of gabapentin once daily on Neuropathic Pain Scale (NPS) score in patients with postherpetic neuralgia (PHN). Pain Med. 2012;13(2):341.

52. Jensen MP, Hsu PH, Vanhove GF. Early pain reduction can predict treatment response: results of integrated efficacy analyses of a once-daily gastroretentive formulation of gabapentin in patients with postherpetic neuralgia. Pain Med. 2012;13(8):1059-66.

53. Once-daily therapy for postherpetic neuralgia. Clinical Advisor. 2011;14(11):31-2.

54. Sang CN, Sathyanarayana R, Sweeney M. Gastroretentive gabapentin (G-GR) formulation reduces intensity of pain associated with postherpetic neuralgia (PHN). Clinical J Pain. 2013;29(4):281-8.

55. Irving GA, Sweeney M. Tolerability and safety of gastroretentive once-daily gabapentin tablets for the treatment of postherpetic neuralgia. Journal of pain research. 2012;5:203-8.

56. Irving G, Carter F, Sondag E, Sweeney M. Tolerability and safety of gabapentin extended-release tablets in the treatment of postherpetic neuralgia. J Am Pharm Assoc. 2011;51(2):253.

57. Irving G, Sondag E, Sweeney M. Tolerability and safety of gabapentin extended-release tablets in the treatment of postherpetic neuralgia Pain Med. 2011;12(3):509.

58. Depomed. Phase 3 Multicenter, Randomized, Double-Blind, Placebo-Controlled Study of the Safety and Efficacy of Gabapentin Extended Release (G-ER) Tablets in the Treatment of Patients With Postherpetic Neuralgia. In: ClinicalTrials.gov [Internet]. Bethesda (MD): National Library of Medicine (US) 2000 [cited 26 May, 2015]. Available from: <https://clinicaltrials.gov/ct2/show/NCT00636636>. NLM Identifier: NCT00636636.

59. Gupta A, Li S. Safety and efficacy of once-daily gastroretentive gabapentin in patients with postherpetic neuralgia aged 75 years and over. Drugs and Aging. 2013;30(12):999-1008.

60. Rauck RL, Irving GA, Wallace MS, Vanhove GF, Sweeney M. Once-daily gastroretentive gabapentin for postherpetic neuralgia: Integrated efficacy, time to onset of pain relief and safety analyses of data from two phase 3, multicenter, randomized, double-blind, placebo-controlled studies. J Pain Symptom Manag. 2013;46(2):219-28.

61. Sweeney M, Sathyanarayana R. Efficacy and tolerability of once-daily gabapentin extended-release (G-ER) for the treatment of postherpetic Neuralgia in patients at least 65 years old. J Gen Intern Med. 2011;26:S161.

62. Serpell MG, Neuropathic Pain Study Group. Reply to Comment on: Serpell et al., gabapentin in neuropathic pain syndromes: a randomised double-blind, placebo controlled trial. Pain. 2003;103(1-2):228.

63. Serpell MP, The Neuropathic Pain Study Group, editors. Gabapentin in neuropathic pain syndromes: a randomised, double‐blind, placebo‐controlled trial (Poster). Fifth International Conference on Mechanisms and Treatment of Neuropathic Pain Annual Meeting; 2002 21‐23 November, 2002; Hamilton, Bermuda.

64. Serpell MG. Gabapentin in neuropathic pain syndromes: a randomised, double-blind, placebo-controlled trial. Pain. 2002;99(3):557-66.

65. Parke-Davis. Research Report: 430-00125. A double blind placebo controlled trial of gabapentin for the treatment of patients exhibiting symptoms of neuropathic pain. Protocol Number: 945-430-306 (NN 026). 2000 5 May 2000.

66. Simpson DA. Gabapentin and Venlafaxine in the treatment of painful diabetic neuropathy. Muscle & Nerve. 2000;23(10):1627.

67. Simpson DA. Gabapentin and venlafaxine for the treatment of painful diabetic neuropathy. Journal of Clinical Neuromuscular Disease. 2001;3(2):53-62.

68. Pérez HE, Sánchez GF. Gabapentin therapy for diabetic neuropathic pain. American Journal of Medicine. 2000;108(8):689.

69. Tamez Pérez HE, Rodríguez Ayala M, Gómez de Ossio MD. Uso de gabapentina en la neuropatía diabética dolorosa [Use of gabapentin on neuropathic diabetic pain]. Med Interna Méx. 1998;14(6):251-3.

70. Depomed. A phase 3 multicenter, randomized, double-blind, placebo-controlled study of the safety and efficacy of gabapentin extended release (G-ER) tablets in the treatment of patients with postherpetic neuralgia. In: ClinicalTrials.gov [Internet]. Bethesda (MD): National Library of Medicine (US) 2000 [cited 6 November, 2015]. Available from: <https://clinicaltrials.gov/ct2/show/NCT00335933>. NLM Identifier: NCT00335933.

71. Rowbotham MC. Gabapentin Extended Release (GER) in the treatment of postherpetic neuralgia: A randomized, placebo-controlled clinical trial. Pain Pract. 2009;9:59-60.

72. Wallace MS, Irving G, Cowles VE. Gabapentin extended-release tablets for the treatment of patients with postherpetic neuralgia: a randomized, double-blind, placebo-controlled, multicentre study. Clin Drug Invest. 2010;30(11):765-76.

73. Yildirim K, Sisecioglu M, Karatay S, Erdal A, Levent A, Ugur M, et al. The effectiveness of gabapentin in patients with chronic radiculopathy. Pain Clinic. 2003;15(3):213-8.

74. Zepeda-Vazquez TC, Hernandez-Santos JR, Tenopala-Villegas S, Martinez-Arellano R, Nunez-Quezada G, Ocampo-Abundes L, et al. Manejo del dolor neuropatico en el paciente diabetico con tramadol via oral comparado con la administracion del mismo asociado a la amitrptilina O Gabapentina. Revista Mexicana de Anestesiología Publicación Oficial de la Sociedad Mexicana de Anestesiología. 2001;1(2):84-7.

75. Safety and efficacy trial of the use of quetiapine fumarate (SEROQUEL®) in the treatment of patients with bipolar depression. In: ClinicalTrials.gov [Internet]. Bethesda (MD): National Library of Medicine (US) 2000 [cited 1 June, 2015]. Available from: <https://clinicaltrials.gov/ct2/show/NCT00060489>. NLM Identifier: NCT00060489 [Internet].

76. Calabrese J, Macritchie KAN, Young AH, Gustafsson U, Paulsson B. The efficacy of Quetiapine monotherapy in bipolar depression: Combined data from the BOLDER and EMBOLDEN studies. Int J Psychiatry Clin Pract. 2009;13(SUPPL. 1):36-7.

77. Eriksson H, Fajutrao L, Gustafsson U, Paulsson B. Quetiapine in the treatment of bipolar depression: improvements in quality of life and functioning in four randomized, placebocontrolled trials. Bipolar Disorder. 2009;11(S1):37.

78. Fajutrao L, Gustafsson U, Paulsson B, Eriksson H. Quetiapine in the treatment of bipolar depression: Improvements in quality of life and functioning in four randomized, placebocontrolled trials. Int J Psychiatry Clin Pract. 2009;13(S1):28-9.

79. Gustafsson U, Fajutrao L. Effect of quetiapine on functioning and quality of life in bipolar depression. Eur Psychiat. 2011;26.

80. Young AH, Calabrese JR, Gustafsson U, Paulsson B, Malhi GS, Bond DJ, et al. The efficacy of quetiapine monotherapy in bipolar II depression: Combined data from the BOLDER and EMBOLDEN studies. Bipolar Disorder. 2009;11(S1):90.

81. Young AH, Macritchie KAN, Calabrese J, Gustafsson U, Paulsson B, Malhi GS, et al. The efficacy of Quetiapine monotherapy in bipolar II depression: Combined data from the BOLDER and EMBOLDEN studies. Int J Psychiatry Clin Pract. 2009;13(S1):37.

82. New data shows the efficacy and good tolerance of Seroquel(R) in bipolar depression. Medecine et Hygiene. 2004;62(2489):1450.

83. Hirschfeld RM, Weisler RH, Raines SR, Macfadden W. Quetiapine in the treatment of anxiety in patients with bipolar I or II depression: a secondary analysis from a randomized, double-blind, placebo-controlled study. J Clin Psychiat. 2006;67(3):355-62.

84. Endicott J, Rajagopalan K, Minkwitz M, Macfadden W. A randomized, double-blind, placebo-controlled study of quetiapine in the treatment of bipolar I and II depression: improvements in quality of life. Int Clin Psychopharm. 2007;22(1):29-37.

85. Endicott J, Rajagopalan K, MacFadden W, Minkwitz M, Gaddy J. Efficacy of quetiapine in improving quality of life in patients with bipolar depression. Bipolar Disorder. 2005;7((Suppl. 2)):P76, 50.

86. Endicott J, Rajagopalan K, Macfadden W, Minkwitz M, Gaddy J. Efficacy of Quetiapine in Improving Quality of Life in Patients with Bipolar Depression. 45th Annual NCDEU (New Clinical Drug Evaluation Unit) Meeting; 2005 June 6 - 9; Boca Raton, FL. 2005:280.

87. Cookson J, Keck PE, Ketter TA, Macfadden W. Number needed to treat and time to response/remission for quetiapine monotherapy efficacy in acute bipolar depression: evidence from a large, randomized, placebo-controlled study. Int Clin Psychopharm. 2007;22(2):93-100.

88. Cookson J, Keck PE, Ketter TA, Macfadden W, Minkwitz MC, Mullen J. Quetiapine in bipolar depression: NNT and time-to-event analyses. Bipolar Disorder. 2005;7(Suppl. 2):27-117.

89. Cookson J, Keck PE, Ketter TA, Macfadden W, Minkwitz MC, Mullen J, editors. Quetiapine in bipolar depression: NNT and time-to-event analyses 158th Annual Meeting of the American Psychiatric Association; 2005 May 21-26; Atlanta, GA; 2005.

90. AstraZeneca. A multicenter, double-blind, randomized, placebo-controlled, double- dummy trial of the use of quetiapine fumarate (SEROQUEL[Registered]) in the treatment of patients with bipolar depression. Study code: 5077US/0049. 2005 28 November 2005.

91. Young AH, Calabrese JR, Gustafsson U, Berk M, McElroy SL, Thase ME, et al. Quetiapine monotherapy in bipolar II depression: Combined data from four large, randomized studies. Int J of Bipolar Disorders. 2013;1(10):1-12.

92. Endicott J, Paulsson B, Gustafsson U, Schiöler H, Hassan M. Quetiapine monotherapy in the treatment of depressive episodes of bipolar I and II disorder: Improvements in quality of life and quality of sleep. J Affect Disorders. 2008;111(2-3):306-19.

93. Thase ME. Quetiapine monotherapy for bipolar depression. Neuropsychiatric Disease & Treatment. 2008;4(1):11-21.

94. Suppes T, Hirschfeld RM, Vieta E, Raines S, Paulsson B. Quetiapine for the treatment of bipolar II depression: analysis of data from two randomized, double-blind, placebo-controlled studies. World J Biol Psychiatry. 2008;9(3):198-211.

95. Weisler RH, Calabrese JR, Thase ME, Arvekvist R, Stening G, Paulsson B, et al. Efficacy of quetiapine monotherapy for the treatment of depressive episodes in bipolar I disorder: a post hoc analysis of combined results from 2 double-blind, randomized, placebo-controlled studies. J Clin Psychiat. 2008;69(5):769-82.

96. MacFadden W, Calabrese J, Suppes T, McCoy R, Minkwitz M, Wilson E, et al., editors. Quetiapine in bipolar I depression: Double-blind, placebo-controlled study. 158th Annual Meeting of the American Psychiatric Association; 2005 May 21-26; Atlanta, GA; 2005 May 21-26; Atlanta, GA.

97. Macfadden W, Calabrese JR, Suppes T, McCoy R, Minkwitz M, Wilson E, et al. Quetiapine in bipolar I depression: doubleblind, placebo-controlled study. Bipolar Disorder. 2005;7 Suppl 2:72.

98. Macfadden W, Calabrese JR, McCoy R, Minkwitz M, Wilson E, Mullen J, editors. Antianxiety effects analysis of quetiapine in bipolar depression. 157th Annual Meeting of the American Psychiatric Association; 2004 May 1-6; New York, NY; 2004.

99. Calabrese JR, Keck PE, Macfadden W, Minkwitz M, Ketter TA, Weisler RH, et al. A randomized, double-blind, placebo-controlled trial of quetiapine in the treatment of bipolar I or II depression. American Journal of Psychiatry. 2005;162(7):1351-60.

100. Calabrese JR, Macfadden W, McCoy R, Minkwitz M, Wilson E, Mullen J, editors. Double-blind, placebo-controlled study of quetiapine in bipolar depression. 157th Annual Meeting of the American Psychiatric Association; 2004 May 1-6; New York, NY; 2004.

101. Quetiapine XR in the Treatment of Comorbid Generalized Anxiety Disorder in Bipolar Depression With or Without Substance Use Disorder. In: ClinicalTrials.gov [Internet]. Bethesda (MD): National Library of Medicine (US) 2000 [cited 1 June, 2015]. Available from: <https://clinicaltrials.gov/ct2/show/NCT00671853>. NLM Identifier: NCT00671853 [Internet].

102. Gao K, Wu R, Kemp DE, Chen J, Karberg E, Conroy C, et al. Efficacy and safety of quetiapine-XR as monotherapy or adjunctive therapy to a mood stabilizer in acute bipolar depression with generalized anxiety disorder and other comorbidities: a randomized, placebo-controlled trial. J Clin Psychiat. 2014;75(10):1062-8.

103. A multicenter, double-blind, randomized, placebo-controlled study to evaluate the efficacy and safety of quetiapine fumarate (SEROQUEL) extended release as monotherapy in the treatment of patients with bipolar depression. In: ClinicalTrials.gov [Internet]. Bethesda (MD): National Library of Medicine (US) 2000 [cited 1 June, 2015]. Available from: <https://clinicaltrials.gov/ct2/show/NCT01256177>. NLM Identifier: NCT01256177 [Internet].

104. Li HF, Gu NF, Zhang HY, Wang G, Tan QR, Yang PD, et al. The efficacy and safety of quetiapine extended release (XR) as mono-therapy in the treatment of Chinese patients with bipolar I or II depression. Bipolar Disorder. 2014;16:65-6.

105. Multicentre, double-blind, randomised, parallel group, placebo controlled, phase 3 study of the efficacy & safety of quetiapine fumarate & paroxetine as monotherapy in adult patients with bipolar depression for 8 weeks & quetiapine in continuation (Abbreviated). In: ClinicalTrials.gov [Internet]. Bethesda (MD): National Library of Medicine (US) 2000 [cited 1 June, 2015]. Available from: <https://clinicaltrials.gov/ct2/show/NCT00119652>. NLM Identifier: NCT00119652 [Internet].

106. Young A, McElroy S, Chang W, Olausson B, Paulsson B, Brecher M. Placebo-controlled study with acute and continuation phase of quetiapine in adults with bipolar depression (EMBOLDEN II). Eur Neuropsychopharmacology. 2008;18(S4):S371-72.

107. Anonymous. EMBOLDEN I and II: quetiapine effective for treating bipolar depression. Brown University Psychopharmacology Update. 2010;21(5):1.

108. AstraZeneca. An international, multi-centre, double-blind, randomised, parallel-group, placebo-controlled, phase III study of the efficacy and safety of quetiapine fumarate (Seroquel [Registered], single oral 300 mg or 600 mg dose) and paroxetine as monotherapy in adult patients with bipolar depression for 8 weeks and quetiapine in continuation treatment for 26 up to 52 weeks [D1447C00134]. Synopsis. 2007.

109. McElroy SL, Weisler RH, Chang W, Olausson B, Paulsson B, Brecher M, et al. A double-blind, placebo-controlled study of quetiapine and paroxetine as monotherapy in adults with bipolar depression (EMBOLDEN II). J Clin Psychiat. 2010;71(2):163-74.

110. A Multicenter, Double-blind, Randomized, Parallel-group, Placebocontrolled, Phase III Study of the Efficacy and Safety of Quetiapine Fumarate (SEROQUEL) Sustained-Release as Monotherapy in Adult Patients With Acute Bipolar Depression. In: ClinicalTrials.gov [Internet]. Bethesda (MD): National Library of Medicine (US) 2000 [cited 1 June, 2015]. Available from: <https://clinicaltrials.gov/ct2/show/NCT00422214>. NLM Identifier: NCT00422214 [Internet].

111. Datto C, Minkwitz M, Nordenhem A, Walker C, Darko D, Suppes T. Effectiveness of the new extended-release formulation of quetiapine as monotherapy for the treatment of acute bipolar depression (trial D144CC00002). Eur Psychiat. 2009;24:S574.

112. Suppes T, Datto C, Minkwitz M, Nordenhem A, Walker C, Darko D. Effectiveness of the extended release formulation of quetiapine as monotherapy for the treatment of acute bipolar depression. J Affect Disorders. 2010;121(1-2):106-15.

113. Hill+Knowlton Strategies. BOLDER II study confirms therapeutic potential of SEROQUEL in bipolar depression. EurekAlert! [21 October 2005]. Available from: <http://www.eurekalert.org/pub_releases/2005-10/hak-bis102105.php>. 2005.

114. Controlled study of the use of quetiapine fumarate in the treatment of patients with bipolar depression. In: ClinicalTrials.gov [Internet]. Bethesda (MD): National Library of Medicine (US) 2000 [cited 1 June, 2015]. Available from: <https://clinicaltrials.gov/ct2/show/NCT00083954>. NLM Identifier: NCT00083954 [Internet].

115. Thase ME. BOLDER II study of quetiapine therapy for bipolar depression. Future Neurology. 2007;2(4):373-7.

116. Goodwin GM. Quetiapine more effective than placebo for depression in bipolar I and II disorder. Evid Based Ment Health. 2007;10(3):82.

117. AstraZeneca. A confirmatory multicenter, double-blind, randomized, placebo-controlled study of the use of quetiapine fumarate (SEROQUEL) in the treatment of patients with bipolar depression [D1447C00135]. 2005.

118. Thase ME, Macfadden W, Weisler RH, Chang W, Paulsson B, Khan A, et al. Efficacy of quetiapine monotherapy in bipolar I and II depression: a double-blind, placebo-controlled study (the BOLDER II study). J Clin Psychopharmacol. 2006;26(6):600-9.

119. Young A, McElroy S, Chang W, Olausson B, Paulsson B, Brecher M. Placebo-controlled study with acute and continuation phase of quetiapine in adults with bipolar depression (EMBOLDEN I). Eur Neuropsychopharmacology. 2008;18(S4):S371-72.

120. Young AH. Erratum: A double-blind, placebo-controlled study with acute and continuation phase of quetiapine and lithium in adults with bipolar depression (Embolden I) (Bipolar Disorders (2008) vol. 10). Bipolar Disorder. 2008;10(3):451.

121. Multi-centre, double-blind, randomised, parallel-group, placebo-controlled, phase 3 study of the efficacy & safety of quetiapine fumarate & lithium as monotherapy in adult patients with bipolar depression for 8 weeks & quetiapine in continuation. In: ClinicalTrials.gov [Internet]. Bethesda (MD): National Library of Medicine (US) 2000 [cited 1 June, 2015]. Available from: <https://clinicaltrials.gov/ct2/show/NCT00206141>. NLM Identifier: NCT00206141 [Internet].

122. Grunze H. Quetiapine is effective in the treatment of adults in the acute phase of bipolar depression. Evid Based Ment Health. 2010;13(3):88.

123. Young AH, McElroy SL, Bauer M, Philips N, Chang W, Olausson B, et al. A double-blind, placebo-controlled study of quetiapine and lithium monotherapy in adults in the acute phase of bipolar depression (EMBOLDEN I). J Clin Psychiat. 2010;71(2):150-62.

124. AstraZeneca. An international, multi-centre, double-blind, randomised, parallel-group, placebo-controlled, phase III study of the efficacy and safety of quetiapine fumarate (Seroquel, single oral 300 mg or 600 mg dose) and lithium as monotherapy in adult patients with bipolar depression for 8 weeks and quetiapine in continuation treatment for 26 up to 52 weeks [D1447C0001]. Synopsis. 2007.
